# Copy Number Change of the NDM-1 Sequence in a Multidrug-Resistant *Klebsiella pneumoniae* Clinical Isolate

**DOI:** 10.1371/journal.pone.0062774

**Published:** 2013-04-29

**Authors:** Tzu-Wen Huang, Te-Li Chen, Ying-Tsong Chen, Tsai-Ling Lauderdale, Tsai-Lien Liao, Yi-Tzu Lee, Chien-Pei Chen, Yen-Ming Liu, Ann-Chi Lin, Ya-Hui Chang, Keh-Ming Wu, Ralph Kirby, Jui-Fen Lai, Mei-Chen Tan, Leung-Kei Siu, Chung-Ming Chang, Chang-Phone Fung, Shih-Feng Tsai

**Affiliations:** 1 Institute of Molecular and Genomic Medicine, National Health Research Institutes, Miaoli, Taiwan; 2 National Institute of Infectious Diseases and Vaccinology, National Health Research Institutes, Miaoli, Taiwan; 3 Division of Infectious Diseases, Taipei Veterans General Hospital, Taipei, Taiwan; 4 Immunology Research Center, Taipei Veterans General Hospital, Taipei, Taiwan; 5 Institute of Genomics and Bioinformatics, National Chung-Hsing University, Taichung, Taiwan; 6 Biotechnology Center, National Chung-Hsing University, Taichung, Taiwan; 7 Institute of Clinical Medicine, National Yang-Ming University, Taipei, Taiwan; 8 Genome Research Center, National Yang-Ming University, Taipei, Taiwan; 9 Department of Life Sciences and Institute of Genome Sciences, National Yang-Ming University, Taipei, Taiwan; 10 Department of Medicine, Chutung Veterans Hospital, Chutung, Hsinchu County, Taiwan; Université d'Auvergne Clermont 1, France

## Abstract

The genetic features of the antimicrobial resistance of a multidrug resistant *Klebsiella pneumoniae* strain harboring *bla*_NDM-1_ were investigated to increase our understanding of the evolution of NDM-1. The strain, KPX, came from a Taiwanese patient with a hospitalization history in New Delhi. Complete DNA sequencing was performed; and the genes responsible for antimicrobial resistance were systematically examined and isolated by library screening. KPX harbored two resistance plasmids, pKPX-1 and pKPX-2, which are 250-kb and 141-kb in size, respectively, with *bla*_NDM-1_ present on pKPX-1. The plasmid pKPX-1 contained genes associated with the IncR and IncF groups, while pKPX-2 belonged to the IncF family. Each plasmid carried multiple antimicrobial resistance genetic determinants. The gene responsible for resistance to carbapenems was found on pKPX-1 and that for resistance to aztreonam was found on pKPX-2. To our surprise, we discovered that *bla*_NDM-1_ exists on pKPX-1 as multiple copies in the form of tandem repeats. Amplification of *bla*_NDM-1_ was found to occur by duplication of an 8.6-kb unit, with the copy number of the repeat varying from colony to colony. This repeat sequence is identical to that of the pNDM-MAR except for two base substitutions. The copy number of *bla*_NDM-1_ of colonies under different conditions was assessed by Southern blotting and quantitative PCR. The *bla*_NDM-1_ sequence was maintained in the presence of the antimicrobial selection; however, removal of antimicrobial selection led to the emergence of susceptible bacterial populations with a reduced copy number or even the complete loss of the *bla*_NDM-1_ sequence. The dynamic nature of the NDM-1 sequence provides a strong argument for judicious use of the broad-spectrum antimicrobials in order to reduce the development and spread of antimicrobial resistance among pathogens.

## Introduction

A novel metallo-β-lactamase, New Delhi metallo-β-lactamase (NDM-1), was first identified in 2008 in a *Klebsiella pneumoniae* strain isolated from a Swedish patient who had received surgery in a New Delhi hospital [1]. Since then, the bacteria harboring NDM-1 coding sequence, *bla*_NDM-1_, have been isolated worldwide in different species of *Enterobacteriaceae*, *Acinetobacter baumannii* and *Pseudomonas aeruginosa* [2], [3], [4], [5], [6], [7], [8]. The global dissemination of these highly resistant bacteria has drawn significant attention and become a major concern among the general public as well as among medical professionals [5], [9]; this is because most of these bacteria are multidrug resistant and remain susceptible *in vitro* to only a limited number of antimicrobials [10], such as polymyxin and tigecycline.

NDM-1 is a broad-spectrum β-lactamase and is able to hydrolyze all β-lactam antimicrobials except aztreonam [1]. The plasmids carrying *bla*_NDM-1_ seem to have a broad host range [5]; and many of them are readily transferred to *E. coli*, which might be one of the reasons for the rapid dissemination of *bla*_NDM-1_ into various *Enterobacteriaceae* [5], [11]. Furthermore, NDM-1 genes are associated with not only other β-lactam but also non-β-lactam resistance determinants that can be co-harbored on the same plasmid [12], [13]. The ease of the transfer of these broad-host-range multidrug-resistant plasmids within the *Enterobacteriaceae* is very worrisome. It is therefore crucial to gain an understanding of the various resistance determinants carried by these plasmids as well as of various other characteristics associated with the transferability of these plasmids.

Shotgun sequencing has been successfully used to explore the genetic backgrounds of bacteria with multidrug resistant phenotypes. For NDM-1 positive isolates, many partial and complete plasmid sequences have been deposited in public databases and published. To date, nine plasmids carrying *bla*_NDM-1_ isolated from different countries have been investigated; they were found to belong to a range of different incompatibility groups (IncL/M [14], IncA/C [15], [16], [17], IncN2 [18], IncFII [19], IncH [20], IncHI1 [21] and untypeable [22]). Complete sequences of these plasmids indicated that although the core regions of *bla*_NDM-1_ share certain similarity, the regions neighboring *bla*_NDM-1_ are quite variable and contain several insertion sequences, which probably reflects multiple genetic modification events. (For a review see Nordmann *et al.* [12].) Furthermore, it has been shown that the remnant of IS*Aba125* found upstream of *bla*_NDM-1_ and the bleomycin resistance gene (*ble*_MBL_) found downstream of *bla*_NDM-1_ are mostly conserved in *Enterobacteriaceae* [13]. Together these findings provide convincing molecular evidence that allows the dissemination and evolution of the *bla*_NDM-1_ sequence to be traced and the possible environmental origin of strains and plasmids to be pinpointed [15].

In this study, we report the complete genetic determinants encoding antimicrobial resistance in a multidrug resistant *K. pneumoniae* strain carrying *bla*_NDM-1_ from a Taiwanese patient. Our new findings, which are based on laboratory experiments, corroborate the clinical observations collected from this silent carrier of a NDM-1-positive isolate; furthermore, the detailed molecular analysis of the NDM-1 region has generated new insights into the dynamic features of the region’s unique structure.

## Materials and Methods

### Patient and Clinical Course

A 38-year-old Taiwanese man was repatriated to Taiwan after undergoing an operation in a medical center in New Delhi, India. This patient had stayed in an Indian intensive care unit for 8 days [23]. *K. pneumoniae* carrying *bla*_NDM-1_ (designated KPX) was isolated from two rectal swab samples of this patient as part of his routine clinical treatment. The identification and the antimicrobial profile of these isolates were fully characterized (Table 1). About one month after the first isolation of the NDM-1 positive *K. pneumoniae*, the Taiwan CDC announced that the bacterium had not been detected in three consecutive samples from rectal swabs [24].

**Table 1:** Minimal inhibitory concentrations (MICs)* for different antimicrobial agents of *K. pneumoniae* KPX, *E. coli* DH10B, and *E. coli* transformants of DNA derived from *K. pneumoniae* KPX.

Agent	KPX		*E. coli* DH10B		ECX-1		ECX-2	
	MIC	Interpretation	MIC	Interpretation	MIC	Interpretation	MIC	Interpretation
Ampicillin	>256	R	4	S	>256	R	>256	R
Piperacillin	>256	R	1.5	S	>256	R	>256	R
Ampicillin/sulbactam	>256	R	3	S	>256	R	24	R
Ticarcillin/clavulanate	>256	R	6	S	>256	R	64	I
Piperacillin/tazobactam	>256	R	2	S	>256	R	6	S
Cefazolin	>32	R	2	S	>32	R	>32	R
Cefuroxime	>256	R	4	S	>256	R	>256	R
Cefoxitin	>256	R	6	S	>256	R	6	S
Cefotaxime	>256	R	0.125	S	>256	R	>256	R
Ceftazidime	>256	R	0.38	S	>256	R	16	S
Ceftriaxone	>256	R	0.094	S	>256	R	192	R
Cefepime	>256	R	0.064	S	>256	R	8	S
Aztreonam	256	R	0.19	S	0.19	S	48	R
Ertapenem	>32	R	0.008	S	>32	R	0.016	S
Imipenem	12	R	0.25	S	>32	R	0.25	S
Meropenem	16	R	0.032	S	32	R	0.032	S
Doripenem	32	R	0.032	S	32	R	0.032	S
Gentamicin	>1024	R	0.5	S	0.75	S	24	R
Amikacin	>256	R	2	S	2	S	4	S
Nalidixic acid	>256	R	1.5	S	1.5	S	3	S
Ciprofloxacin	>32	R	0.004	S	0.004	S	0.19	S
Levofloxacin	>32	R	0.008	S	0.008	S	0.064	S
Trimethoprim/sulfamethoxazole	>32	R	0.0147	S	>32	R	>32	R
Chloramphenicol	>256	R	4	S	3	S	4	S
Tetracycline	>256	R	1.5	S	1.5	S	32	R
Minocycline	12	I/R	0.5	S	0.75	S	0.75	S
Tigecycline	1	S	0.19	S	0.19	S	0.19	S
Polymyxin E	0.38	S	≤0.064	S	≤0.064	S	≤0.064	S

* MIC interpretations are based on Clinical and Laboratory Standards Institute (CLSI) breakpoints (CLSI M100-S21, [28]), except for polymyxin E and tigecycline, which are based on EUCAST (http://www.eucast.org/clinical_breakpoints/) breakpoints.

### Bacterial Identification and Antimicrobial Susceptibilities Tests

The cultured samples were inoculated onto MacConkey agar plates (BBL, Becton Dickinson Microbiology Systems, Cockeysville, MD) containing imipenem (1 µg/ml). Bacteria that grew on these plates were tested for the presence of carbapenemases by the Modified Hodge test (MHT) [25]. Bacteria that gave a positive result for the MHT were screened for the presence of *bla*_NDM-1_ by PCR using primers targeting the *bla*_NDM-1_ gene (GenBank accession number FN396876.1). Identification of KPX was performed by conventional biochemical methods and by 16S rRNA sequence analysis [26]. Multilocus sequence typing (MLST) was used to identify the clonality of the isolate [27]. Minimal inhibitory concentrations of various antimicrobials were determined by the reference broth microdilution method using custom-designed panels (Sensititre, Trek Diagnostics, West Essex, England) following the guidelines of the Clinical and Laboratory Standards Institute [28] and by the E-test following the manufacturer’s recommendations.

### DNA Sequencing and Gene Annotation

To identify the genetic determinates of antimicrobial resistance we performed shotgun sequencing of the KPX plasmids. The plasmids were extracted using a Qiagen large-construct kit (Qiagen, Valencia, CA, USA). Shotgun sequencing was performed to a 160-fold coverage using a Genome Sequencer Junior instrument (Roche) using a library consisting of 800-bp shotgun fragments. Additional small-size (3∼4-kb and 5∼8-kb inserts) shotgun libraries were constructed from the plasmid DNA [29], [30] and then sequenced to 2.3-fold coverage of the targets using ABI3730xl automated capillary electrophoresis sequencers (Applied Biosystems, Foster City, CA, USA). Taking the linking information of paired sequences, contigs were inspected manually and reassembled, using the Consed visualization and editing system [31]. Two circular DNA sequences (pKPX-1 and pKPX-2) were eventually assembled; and the orientation and position of the constituent contigs were confirmed by matrix PCR and by sequencing selected cloned *Bam*HI and *Eco*RI fragments.

Open reading frames (ORF) were predicted based on the start and termination codons. Sequence homology was searched against the GenBank database using the BLAST program (http://blast.ncbi.nlm.nih.gov/). Manual annotation was carried out throughout the sequence; and any discrepancies were curated.

### Molecular Analysis

KPX was cultured in 200-ml Luria-Bertani (LB) broth for overnight at 37°C. Bacterial cells were harvested by centrifugation and plasmids were extracted as for DNA sequencing. To separate different plasmids, 0.6 µg plasmid DNA from KPX was electroporated into ElectroMAX™ DH10B™ T1 Phage-Resistant electrocompetent *E. coli* cells (Invitrogen, Carlsbad, CA, USA) following the manufacturer’s instructions. Two transformants selected from the Luria agar plate containing either 4 µg/ml imipenem (ECX-1) or 100 µg/ml ampicillin (ECX-2) were confirmed for the presence of the NDM-1 gene by antimicrobial susceptibility test and PCR.

To verify the DNA sequence assembly, plasmid DNA from KPX, ECX-1 and ECX-2 digested with different restriction enzymes was separated by either conventional agarose gel electrophoresis or pulsed field gel electrophoresis (PFGE) using a CHEF-DEII system (Bio-Rad Laboratories, Hercules, CA, USA). The PGFE was carried out using a 1% agarose gel and 0.5X TBE buffer at 6 volts per centimeter and 14°C for 16 hours, with a switch time of 1–25 seconds and a rotated angle of 120 degrees. To investigate the genetic organization of the *bla*_NDM-1_ locus, Southern blotting was performed using DIG-labeled probes (Roche Diagnostics, Mannheim, Germany).

### Conjugation Test

To test the conjugal ability of KPX plasmids, the original *K. pneumoniae* strain, KPX, was mated to an *E. coli* recipient, J53, which is resistant to sodium azide, at 25°C, at 30°C, and at 37°C using overnight culture. The mating mixture was then resuspended in LB broth and spread onto plates containing both 100 µg/ml sodium azide and 100 µg/ml ampicillin. After overnight culture, the grown colonies were tested for their resistance to either imipenem or gentamicin.

### Isolation of Antimicrobial Resistance Associated Sequences

In order to identify the genetic determinants for amikacin or chloramphenicol resistance, two shotgun libraries with average insert sizes of 3.5-kb and 6-kb were constructed. In total, 1920 colonies were tested for growth in the presence of amikacin or chloramphenicol medium at different concentrations of antibiotic (16, 32, and 64 µg/ml). Colonies that grew on the individual selection medium were picked. Thirty clones conferring amikacin resistance and twenty clones conferring chloramphenicol resistance were sequenced from both ends of their inserts.

### Imipenem Selection of bla_NDM-1_

Bacterial KPX was spread onto LB agar plates with different concentrations of imipenem. After overnight incubation, multiple single colonies from each plate were inoculated into LB broth that contained the same concentration of imipenem and grown at 37°C for 4–7 hours. Genomic DNA derived from each colony was extracted by Wizard DNA purification kit (Promega, Madison, WI, USA). To estimate the relative copy number of the NDM-1 gene, two sequences that could be amplified from pKPX-1 – namely *pcoB* (pcoB-F: TGCCATGCAGATGCCAGCAGAT; pcoB-R: ATCCGACCGTTGCCATTCCAG) and *bla*_NDM-1_ (NDM_qPCR_F: TGCCCAATATTATGCACCCGG; NDM_qPCR_R: CGAAACCCGGCATGTCGAGA) – were designed. Quantitative PCR reactions were carried out using 5 µl KAPA SYBR® FAST ABI Prism® 2X qPCR Master Mix (Kapa Biosystems, Woburn, MA, USA), 2 ng genomic DNA as template, and 2 pmole of each primer in a 10 µl reaction. The Ct values of each sample was measured under appropriate PCR conditions (preheat at 50°C for 2 min; denature at 95°C for 20 sec; 40 amplified cycles at 95°C for 3 sec and 60°C for 20 sec) on an ABI PRISM 7900HT. The relative fold change of *bla*_NDM-1_ for each sample was normalized against a housekeeping gene (*pcoB*) by using the comparative Ct method [32].

### Ethics Statement

This research did not involve human subjects. The bacterial samples used in this study were previously gathered as part of routine clinical work. The clinical information presented in this paper on the patient has been published previously [23], [24]. We consulted NHRI’s Institutional Review Board regarding whether this study would require review and none was required.

## Results

### Antimicrobial Susceptibility Testing

Scrutiny of a returnee from India identified that the individual was positive for *K. pneumoniae* carrying *bla*_NDM-1_ [23]. The *K. pneumoniae* belonged to ST type 11. Table 1 summarizes the resistance phenotypes of this isolate (designated KPX) for several classes of antimicrobials. Similar to other NDM-1 positive enteric bacteria, this clinical isolate is resistant to all tested antimicrobials except polymyxin E and tigecycline. To facilitate sequence assembly and to map the genetic determinants of antimicrobial resistance, two types of plasmid subclones were obtained by *E. coli* transformation (Table 1). The first, ECX-1, was resistant to imipenem; and the other, ECX-2, was susceptible to imipenem but resistant to ampicillin. ECX-2 also showed susceptibility to quinolones. The plasmids carried by the two transformants were named pECX-1 and pECX-2, respectively.

### Molecular and Genomic Analysis

**Figure 1:**
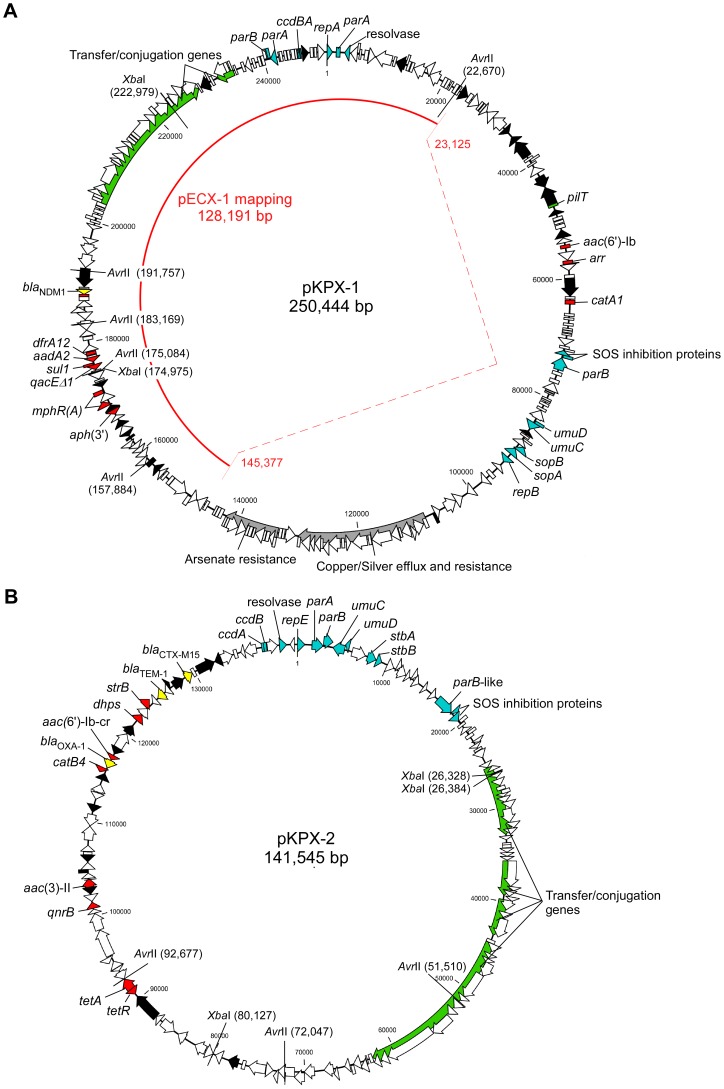
Sequence analysis of KPX plasmids. Two circular sequences are shown for the organization of pKPX-1 (**A**) and pKPX-2 (**B**). Mapping shotgun sequencing reads of pECX-1 to the pKPX-1 is indicated by the red half-circle. A large part of the plasmid, corresponding to the nucleotide positions 23,125 to 145,377 of pKPX-1, was not found in pECX-1. Only the part on the left side, totaling 128,191-bp, is retained. Two genes encoding chloramphenicol and amikacin resistance were identified by functional library screening. Their positions in the deleted region are indicated. Nucleotides are numbered according to the replication origin. Genes are color coded: yellow, β-lactamase; red, antimicrobial resistance associated; blue, plasmid replication and partitioning; black, transposases or IS elements; and white, other coding sequences of miscellaneous features. The arrows on the open reading frames (ORFs) indicate the gene orientation. Gene clusters involved in gene transfer or mobility are marked in green. *Xba*I and *Avr*II restriction sites are shown inside the circle.

**Figure 2:**
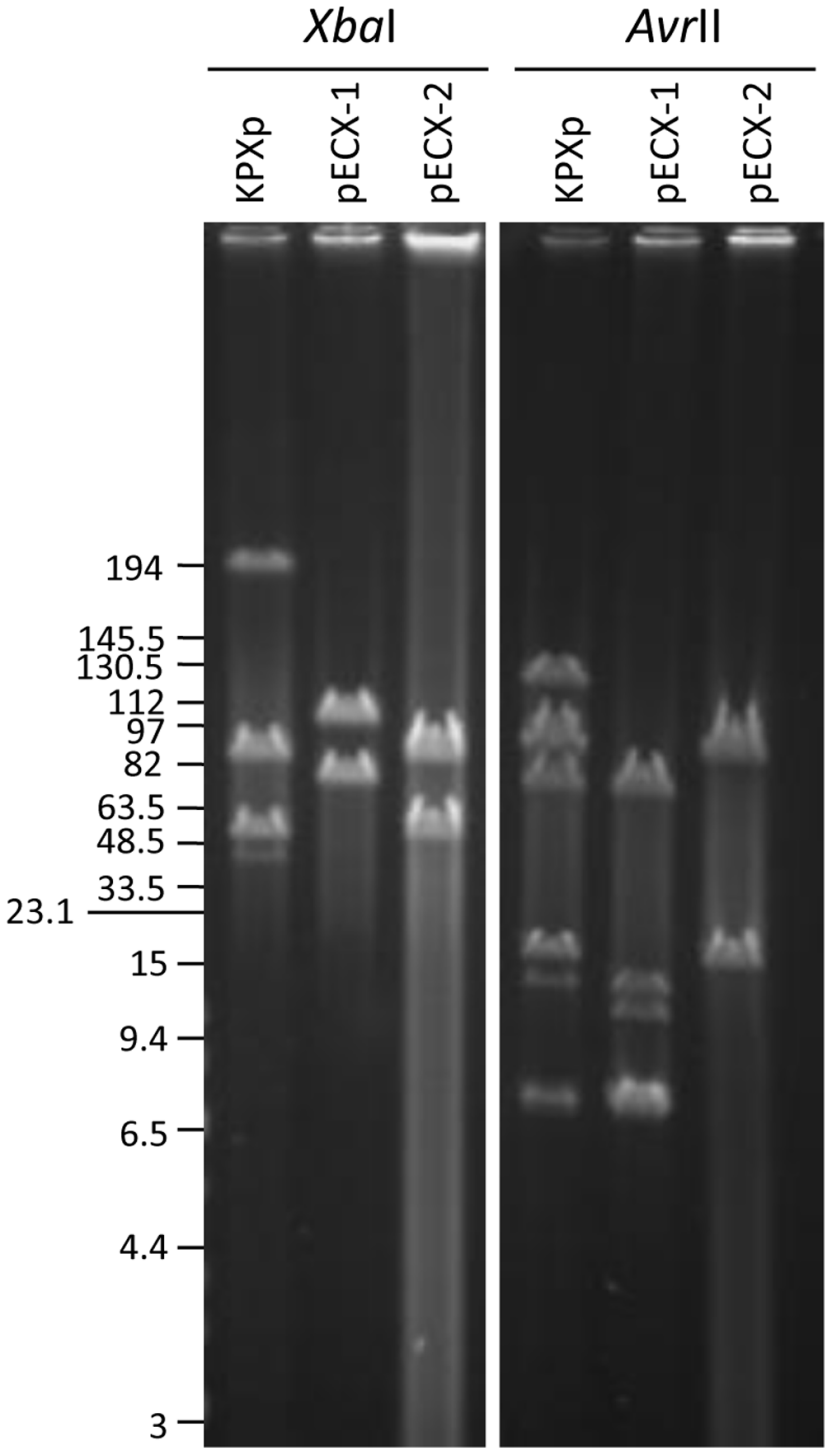
Restriction analysis of the KPX plasmids by PFGE. Plasmid DNA isolated from the KPX isolate was analyzed using pulsed field gel electrophoresis (PFGE). The restriction patterns of the plasmid DNA were compared to those of pECX-1 and pECX-2 in *Escherichia coli*. The size of the DNA markers is shown in kilobases (kb) on the left side.

We applied a combination of genetic and genomic methods, including 454 sequencing technology [33] and library screening [34], to obtain a comprehensive in-depth view of the antimicrobial resistance of this particular isolate. A total of 165,600 reads (64 megabases) generated from the 454 GS Junior as well as 1,152 paired-end Sanger reads (910 kilobases) were assembled using the Newbler sequence assembler (version 2.3). As shown in Fig. 1, the two circular plasmids from the KPX isolate were 250,444-bp (GenBank accession number AP012055) and 141,545-bp (GenBank accession number AP012056) in size; they were designated pKPX-1 and pKPX-2, respectively. Pulse field gel electrophoresis (PFGE) was used to analyze the plasmid DNA of the *E. coli* transformants; and their restriction patterns with *Xba*I or *Avr*II were compared to the full plasmid profile of the KPX isolate. The plasmids from KPX, when digested with *Xba*I, generated four fragments of approximately 202-kb, 88-kb, 54-kb, and 48-kb in size. Two bands, 202-kb and 48-kb, were derived from pKPX-1, while the other two bands, 88-kb and 54-kb, were derived from pKPX-2. In contrast, the plasmid (pECX-1) from the imipenem-resistant *E. coli* transformants gave two *Xba*I fragments of 108-kb and 80-kb, which suggests that there was a gross rearrangement of pKPX-1 to create pECX-1 (Fig. 1A and Fig. 2). This interpretation was confirmed by the results obtained when the plasmid was digested with *Avr*II.

To delineate the structural difference between pKPX-1 and pECX-1, we performed shotgun sequencing of pECX-1 and mapped the sequencing reads to pKPX-1. As shown in Fig. 1A, approximately 122-kb (nucleotide position 23,125 to 145,377) from the pKPX-1 was absent in the pECX-1 sequence. A 24-bp direct repeat was found at the ends of the deletion fragment; this sequence, 5′-TGATATTTAACATAAAACGCATTA-3′, may have allowed homologous recombination to take place.On the basis of sequence annotation, both pKPX-1 and pKPX-2 contain genes for conjugation functions. However, it was experimentally determined that while pKPX-2 is a conjugative plasmid, pKPX-1 appears to be non-conjugative. Seventy-seven transconjugants, which were selected from plates containing both sodium azide and ampicillin, were tested for resistance to imipenem (targeting pKPX-1) or gentamicin (targeting pKPX-2). All 77 transconjugants were resistant to gentamicin but not to imipenem. Colony PCR targeting for *qnrB* and *aac(6′)-Ib* was performed to confirm the transfer of pKPX-2. Plasmids extracted from two transconjugants were digested with *Avr*II and analyzed by PFGE. The restriction pattern of these plasmids was the same as the pattern of pKPX-2 (data not shown).

### Genetic Determinants of Antimicrobial Resistance

From assembled sequences, we predicted a total of 215 genes that were encoded by the pKPX-1 plasmid and 132 genes that were encoded by the pKPX-2 plasmid. Table 2 summarizes the genes among these that are implicated in resistance to commonly used antimicrobials.

**Table 2:** Antimicrobial resistance determinants in pKPX-1 and pKPX-2.

Antimicrobials	pKPX-1	pKPX-2	KPX chromosome
β-lactams	β-lactamases		
Penicillins	NDM-1	TEM-1	
		CTX-M-15	
		OXA-1	
Narrow-spectrum cephalosporins	NDM-1	TEM-1	
		CTX-M-15	
		OXA-1	
Extended-spectrum cephalosporins	NDM-1	CTX-M-15	
Carbapenem	NDM-1		
Aztreonam		CTX-M-15	
Aminoglycosides	Aminoglycoside-modifying enzymes/16S rRNA methyltransferases		
Amikacin	AAC(6′)-Ib^†^	AAC(6′)-Ib-cr	
	Rmt^†^		
Kanamycin/Neomycin	APH(3′)-I		
Gentamicin		AAC(3)-II	
Streptomycin	ANT(3″)-I	APH(3″)-Ib	
Quinolone and fluoroquinolones		QnrB	GyrA, S83I
		AAC(6′)-Ib-cr	ParC, S80I
Tetracycline		TetA	
Chloramphenicol	CatA1^†^	CatB4*	
Rifampicin	ARR-2^†^		
Trimethoprim/sulfamethoxazole	DHPS (*sul1*)	DHPS (*sul2*)	
	DHFRXII		
Erythromycin	Mph(A)		

* Nonfunctional; ^†^ Deleted in pECX-1.

Abbreviations: AAC, aminoglycoside acetyltransferase; APH, aminoglycoside phosphotransferase; ANT, aminoglycoside nucleotdyltransferase; Rmt, 16S rRNA methyltransferase; Qnr, quinolone resistance protein; TetA, tetracycline efflux protein; Cat, chloramphenicol acetyltransferase; ARR-2, rifampin ADP-ribosyltransferase; DHPS, dihydropteroate synthase; DHFR, dihydrofolate reductase; Mph, macrolide phosphotransferase.

#### β-lactams

**Figure 3:**
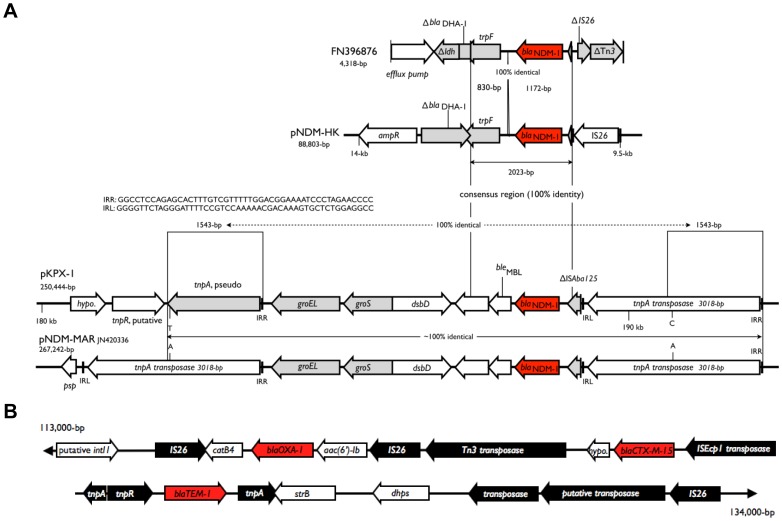
Organization of the *bla*_NDM-1_ sequences and other β-lactamase genes. (**A**) Comparable flanking sequences of *bla*_NDM-1_ in pKPX-1 and those reported for the *K. pneumoniae* ST type 14 sequence (GenBank accession number FN396876) from India and pNDM-MAR of a *K. pneumoniae* ST type 15 from Morocco [21], and of pNDM-HK, an *E. coli* isolate from Hong Kong [14]. The colors are red, *bla*_NDM-1_; gray, pseudogenes; and white, others. The abbreviations are hypo, hypothetical protein; pseudo, pseudogenes; *ldh*, L-lactate dehydrogenase pseudogene; *trpF*, phosphoribosyl anthranilate isomerase pseudogene; *bla*_DHA-1_, AmpC β-lactamase *bla*_DHA-1_ pseudogene; and *psp*, phage shock protein operon transcriptional activator. The 1543-bp identity between the *tnpA* transposase and the incomplete *tnpA* pseudogene is shown. (**B**) Two regions that contain three β-lactamase genes – *bla*_OXA-1_, *bla*_TEM-1_, and *bla*_CTX-M-15_ – are shown. The ORFs encoding the β-lactamases are colored red. Transposons or insertion sequence-related genes are in black. Other genes are in white. The *intI1* gene is a putative integrase. The *catB4* gene encodes a chloramphenicol resistance acetyltransferase. The *aac(6′)-Ib* gene encodes an aminoglycoside resistance 6′-acetyltransferase type Ib. The *dhps* gene encodes a dihydropteroate synthase, which provides sulfonamide resistance. The *strB* gene encodes a streptomycin resistance protein B.

Annotation of the plasmid sequences from the KPX isolate revealed four β-lactamase genes: *bla*_NDM-1_ on pKPX-1, and *bla*_CTX-M-15_, *bla*_TEM-1_, and *bla*_OXA-1_ on pKPX-2. All these β-lactamase genes are adjacent to or flanked by insertion sequences (Fig. 3). When a comparison between pKPX-1 and FN396876, the first reported NDM-1 DNA fragment, was carried out it was found that a 1172-bp region, including the 813-bp of the *bla*_NDM-1_ ORF, is 100% identical. Upstream of the *bla*_NDM-1_, sequence homology stops at an IS*30*-family transposase pseudogene region. On the other side, an 830-bp region was also found to be identical between the two sequences. This colinearity downstream between FN396876 and the pKPX-1 sequence is disrupted by a 21-bp sequence; and this addition between the two conserved sequences allowed the annotation of a bleomycin-resistance gene (61% identity in amino acid sequence of a bleomycin resistance protein from *Caulobacter crescentus* CB15, GenBank accession number NP_421581.1) in pKPX-1. The 813-bp common sequence ends with an extra *bla*_DHA-1_ pseudogene present in FN396876 (Fig. 3A).

#### Aminoglycosides

Genes predicted to confer resistance to aminoglycosides on pKPX-1 are *rmt*, *aac(6′)-Ib*, *aph(3′)-I*, and *aadA2*. On pKPX-2, the genes predicted to be responsible for aminoglycosides resistance are *aac(6′)-Ib-cr*, *aac(3)-II*, and *strB*. To confirm experimentally that the pKPX plasmids were responsible for resistance to the aminoglycosides, we screened shotgun clones using amikacin selection and identified those that grew in the presence of the antimicrobial. Almost all of the selected *E. coli* transformants carried pieces of DNA fragments from pKPX-1 and these sequences were absent from pECX-1. Interestingly, these fragments overlapped and clustered with *aac(6′)-Ib* and *rmt* (Fig. S1). Thus, deletion of the pKPX-1 sequence to give pECX-1 caused the loss of a number of antimicrobial resistance genes. A small number of amikacin-resistant clones were found to contain the sequence of the *aac(6′)-Ib-cr* genes from pKPX-2 (Fig. S1).

#### Quinolones

Two plasmid-mediated quinolone resistance determinants, aminoglycoside acetyltransferase *aac(6′)-Ib-cr* and *qnrB*, which presumably confer reduced susceptibility to fluoroquinolones [35], were found to be present on pKPX-2. The fact that both ECX-1 and ECX-2 are susceptible to quinolones indicates that the KPX chromosome has mutations affecting the target genes of the antimicrobials. Indeed we found mutations in the *gyrA* (Ser83Ile) and *parC* (Ser80Ile) genes, both of which are chromosomal in nature.

#### Tetracycline, erythromycin, and chloramphenicol

We identified *tetA* [36] as a determinant of tetracycline resistance and a gene similar to *mph(A)* [37], which encodes macrolide 2'-phosphotransferase and confers erythromycin resistance. In addition, three genes were identified as possibly able to confer chloramphenicol resistance. These are *catA1*, together with a putative chloramphenicol acetyltransferase gene (putative *cat*) on pKPX-1, and *catB4* on pKPX-2. Since neither ECX-1 nor ECX-2 was resistant to chloramphenicol, we screened shotgun libraries to isolate the responsible sequences from pKPX-1 and pKPX-2. This yielded, respectively, seven and eight clones spanning the *catA1* gene and the putative *cat* that were resistant to chloramphenicol, with minimal inhibitory concentration in the range of 32–64 µg/ml (Fig. S2). The putative *cat* gene encodes a 23.7-kDa protein that is 73% identical to the chloramphenicol acetyltransferase from *Agrobacterium tumefaciens* (GenBank accession number NP_355927.1) at the amino acid level. These two genes, which are presumably responsible for the chloramphenicol resistance of KPX together with *aac(6′)-Ib* and *rmt*, were lost in pECX-1 as a result of deletion (Fig. 1A).

### Comparative Analysis with other NDM-1 Positive Plasmids

Recently, the complete sequencing of many NDM-1 carrying plasmids isolated from *Enterobacteriaceae* has shown that these plasmids belonged to a variety of different incompatibility groups, which is consistent with their wide bacterial host range. The two replication proteins encoded by pKPX-1 belonged to the IncR and IncF groups, while pKPX-2 is a member of the IncF family. To gain insight into the evolution of the *bla*_NDM-1_ genetic element, the coding and flanking sequences of *bla*_NDM-1_ from pKPX-1 were compared to those of the corresponding regions of representative plasmids. As shown in Fig. 3A, only the core sequence of the *bla*_NDM-1_ region is conserved. The right boundary of the homologous sequences starts upstream of the *bla*_NDM-1_ gene near the truncated IS*Aba125* region (position 188,934 to 189,093), while the left boundary ends at variable sites ranging from a *groEL* pseudogene to a bleomycin-resistance gene (*ble*_MBL_, position 183,741 to 187,837). This observation is consistent with findings published in a recent report [13]. Among the reported sequences, pKPX-1shared a long stretch of identical sequence in the NDM-1 region with that of pNDM-MAR [20], an IncH plasmid carried by a *K. pneumoniae* isolate from Morocco.

### Amplification of the bla_NDM-1_ Gene

The region neighboring *bla*_NDM-1_ is variable and filled with insertion sequences that reflect multiple genetic modification events. How *bla*_NDM-1_ arose and is mobilized among the *Enterobacteriaceae* is still not totally clear. However, we have discovered the *bla*_NDM-1_ region of pKPX-1 has a unique structure. As shown in Fig. 3A, two identical 1543-bp sequences flank the far ends of the *bla*_NDM-1_ region. The 1543-bp sequence on the right side is part of a Tn*3*-like IS element, as marked by TnpA transposase and its flanking inverted repeats (IRR and IRL). Thus, the *bla*_NDM-1_ segment could possibly be mobilized as a composite transposon. Alternatively, the 1543-bp repeat at both sides of the approximately 8.6-kb *bla*_NDM-1_ region may serve as a target for homologous recombination. Note that pNDM-MAR also contains this unique structure and also has an intact *tnpA* transpoase gene in the left flanking sequence (Fig. 3A). Only a 2-bp difference is present across the 10,273-bp segment (from position 182,056 to 192,328 of pKPX-1) that is shared between the two plasmids.

**Figure 4:**
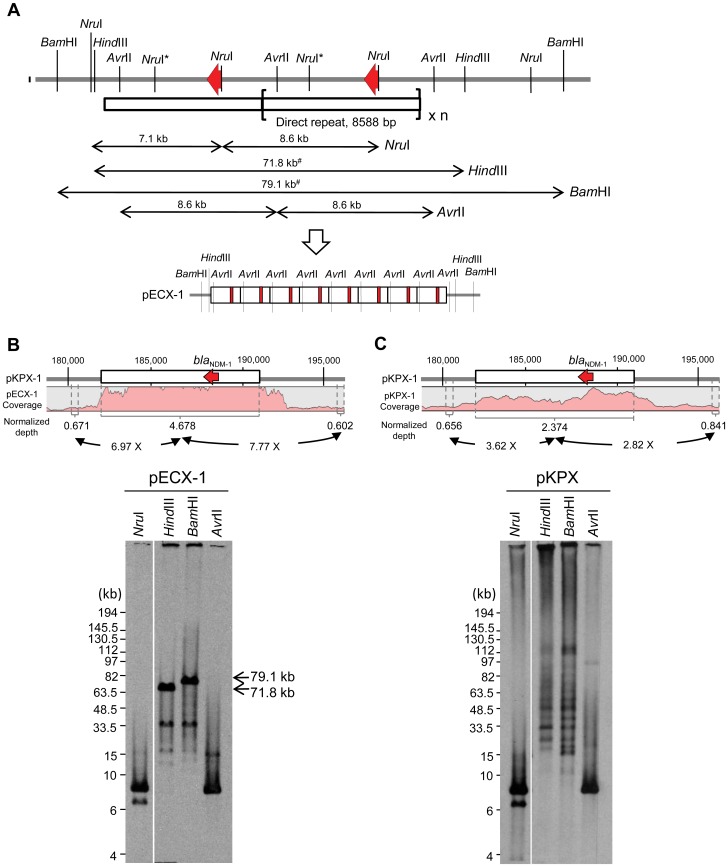
Tandem duplication of the *bla*_NDM-1_ gene in pKPX-1 and pECX-1. (**A**) Diagrammatic representation of the analysis of *bla*_NDM-1_ copy number by Southern blot. The probe is shown with a red arrow, and the tandem duplication of the 8588-bp repeat is indicated by the bracket. The asterisks indicate the methylated *Nru*I sites. The sizes of *Bam*HI or *Hind*III digested fragments depend on the copy number of the repeat. The pound sign indicates 79.1 kb and 71.8 kb for *Bam*HI and *Hind*III restrictions, respectively, when there are 8 copies of the tandem repeat, as in the case of pECX-1. (**B**) Sequencing read distribution and Southern analysis of the *bla*_NDM-1_ region for pECX-1. The upper panel shows the relative coverage depth of the repeat region and its flanking sequences. The average coverage of *bla*_NDM-1_ is 7–8 fold of those sequences of the immediately adjacent regions, suggesting that there are eight copies of the repeat. As shown in the lower panel, Southern analysis confirms this model of tandem duplication. (**C**) *Bla*_NDM-1_ copy number variation detected by the Southern analysis. Sequence depth of the region revealed an average of 3–4 copies of the repeat sequence in pKPX. *Bam*HI and *Hind*III digestion gave a series of ladder bands, corresponding to different copy numbers of the repeat. By contrast, *Avr*II and *Nru*I both deliberated a single major band of 8.6 kb, representing the unit length of the tandem repeats.

It appears that this 8.6-kb unit containing the *bla*_NDM-1_ sequence exists in multiple copies as tandem repeats. By counting the read number across the pKPX-1 and pECX-1 plasmids, we found a much deeper coverage for the NDM-1 region than for the flanking sequences, indicating there was amplification of the NDM-1 coding sequence. To further characterize the organization of the cassette, we conducted Southern analysis of the NDM-1 coding region using several different restriction enzymes (Fig. 4A). As shown in Fig. 4B and 4C, *Avr*II and *Nru*I each generated one major discrete band from pKPX-1 and pECX-1 plasmid DNA. On the other hand, *Hind*III and *Bam*HI generated one major band of 71.8-kb and 79.1-kb, respectively, from pECX-1 (Fig. 4B) but multiple bands for pKPX-1 (Fig. 4C). In both plasmids, the sizes of *bla*_NDM-1_ sequence positive bands produced by *Hind*III and *Bam*HI digest are bigger than those predicted by the assembled sequence. These results demonstrate that there is multiplication of the 8.6-kb cassette. Furthermore, the tandem repeats in pKPX-1 appear to vary across different subpopulations of the cultured bacteria, as a ladder pattern of bands indicates the existence of bacteria carrying a different copy number of the repeats. By way of contrast, the pECX-1 plasmid is relatively homogenous and seems to have a fixed repeat number. We estimate there are eight copies of the cassette present in pECX-1 (Fig. 4B).

Taking together the sequencing and molecular analysis data, we conclude that there is amplification of the NDM-1 coding sequence in the KPX isolate. Moreover, the NDM-1-positive plasmids of the bacteria showed genetic heterogeneity in the *bla*_NDM-1_ copy number.

### Copy Number of the 8.6-kb Repeat under Imipenem Selection

**Figure 5:**
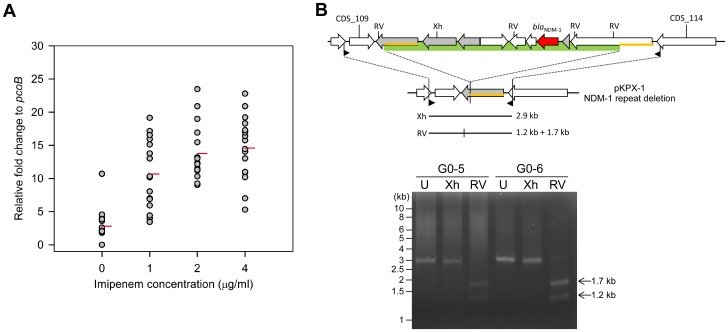
Copy number change of the *bla*_NDM-1_ gene. (**A**) A dot plot of the copy numbers of *bla*_NDM-1_ from colonies selected at four different concentrations of imipenem (0, 1, 2, and 4 µg/ml). The y-axis is the relative fold change of *bla*_NDM-1_ to the *pcoB* gene. Each condition contained 16 individual isolates, which were analyzed by quantitative PCR (qPCR) in triplicate. The red line indicates the mean of fold change under each condition. (**B**) Two isolates (G0–5 and G0–6) with extremely low *bla*_NDM-1_ relative to *pcoB* from the zero imipenem condition were analyzed for the junctional sequences of the 8.6-kb repeat. The diagram shows the abbreviated structure and expected fragment size of *Eco*RV (RV) or *Xho*I (Xh) digestion when the 8.6-kb repeat is deleted. The filled black triangles indicate the positions of the PCR primers (NDMjuc-F: GCTCTCTACGCTGCCTTGCAGG and NDMjuc-R: GTACCTGGAGACAGTGGGCAAG). The green box indicates the 8.6-kb repeat. The yellow lines indicate the 1543-bp identical region, as described in Figure 3. The bottom image shows the uncut (U), *Eco*RV, or *Xho*I digested PCR products of G0–5 and G0–6.

To investigate whether *bla*_NDM-1_ copy number is affected by antimicrobial selection, colonies isolated from cultures at different imipenem concentrations were analyzed for their *bla*_NDM-1_ structure by a real-time PCR assay. As shown in Fig. 5A, the copy number varied significantly when KPX was cultured in the absence of imipenem, while the *bla*_NDM-1_ gene copy was consistently higher as the imipenem concentration was increased (the mean expression levels relative to a housekeeping gene, *pcoB*, were 2.8, 10.7, 13.8, and 14.6 at 0 µg/ml, 1 µg/ml, 2 µg/ml, and 4 µg/ml, respectively). This finding was supported in a separate experiment using imipenem concentration ranging from 0 to 256 µg/ml (data not shown). Taken together, our results indicated that the *bla*_NDM-1_ gene was maintained high in the plasmid when the imipenem concentration was 2 µg/ml and above, but that the KPX bacteria lost the imipenem resistance gene when selection pressure was lifted.

To understand the structural changes that occur in the NDM-1 region isolated from these imipenem sensitive isolates at a molecular level, a 2.9-kb DNA fragment was amplified by PCR using a unique primer pair that annealed to the sequences outside the NDM-1 repeat unit (Fig. 5B). The size of amplified DNA and its restriction pattern after *Xho*I and *Eco*RV digestion were the same as predicted (Fig. 5B). Sequencing this amplified DNA fragment confirmed that the entire 8.6-kb repeat unit was removed via the flanking 1.5-kb tandem repeats (Fig. S3).

## Discussion

In the present study, we have fully analyzed the plasmid sequences present in this bacterial isolate, from which it can be concluded that the antimicrobial resistance determinants of a *bla*_NDM-1_ carrying *K. pneumoniae* strain are distributed over three separate replicons, namely the chromosome and two plasmids (Table 2). The antimicrobial resistance determinants were uncovered by elucidating their sequences in the context of antimicrobials susceptibility profiling; and these findings were corroborated by library screen and isolation of the drug-resistant sequences. We found that the *K. pneumoniae* genetic system has the potential to acquire, alter, and transfer drug resistance genes. Such transmissibility and plasticity underpin the prevalence and diversity of NDM-1 positive *Enterobacteriaceae* populations. The present findings should help with the creation of measures to reduce the development and spread of antimicrobial resistance among bacterial pathogens.

The reduction in copy number and loss of the *bla*_NDM-1_ gene in the absence of strong imipenem selection suggests that either the high copy number of this gene or the presence of the gene itself has a negative impact on the fitness of the *Klebsiella* bacterium that contains this sequence, as discussed by Solé *et al*. [38]. This supports the idea that it is logical and important to control antimicrobial resistance in a hospital setting by reducing the use of broad-spectrum antimicrobials. Unnecessary use of antimicrobials will favor bacterial strains that gain advantage under such manmade selection pressure. This is not only true for carbapenems but also for ciprofloxacin. In this context, the *K. pneumoniae* host harboring the *bla*_NDM-1_ gene was found to also have chromosomal mutations in *gyrA* and *parC*.

From an evolutionary point of view, gene amplification is important for bacteria to cope with antibiotic selection. Among bacteria, just as with other kingdoms of life, copy number change provides a way of generating genetic variability for adaptation to altered growth conditions [39]. Amplification of plasmid-borne resistance genes conferring the high-level resistance to antibiotics such as β-lactams or tetracycline, etc., has been reported in a range of different bacteria [40], [41], [42], [43], [44], [45]. The dosage of resistance genes is flexible and hinges upon concentration of the antibiotics. The proposed mechanism of amplification typically involves the insertion sequence (IS) elements associated with the resistance genes [43], [44]. Indeed, in our case the *bla*_NDM-1_ sequence was flanked by a 1543-bp direct repeat, which was part of a Tn*3*-like element. This mobile element (containing *tnpA*) is well preserved in pNDM-MAR [20] on the two sides of the NDM-1 region (Fig. 3A). Considering the similarity of the NDM-1 region between pKPX-1 and pNDM-MAR, it is possible that they share a common origin for the *bla*_NDM-1_ sequence, presumably from India.

Interestingly, the copy number of *bla*_NDM-1_ was fixed after pKPX was transformed into a *recA*-defective *E. coli* host, whereas the copy number varied in the original KPX strain. It suggests that dynamic change of the *bla*_NDM-1_ region is RecA-dependent. This point is consistent with a previous report [46]. Of note, high resistance to carbapenems mediated through amplification of *bla*_OXA-58_ has been reported [44]. To our knowledge, this is the first clinical case showing amplification of the *bla*_NDM-1_ gene in the many reports of sequenced NDM-1-positive plasmids. Finally, gene amplification is unstable and usually detected when bacteria are grown in the presence of antibiotics under laboratory conditions [39]. Our data indicate this clinical KPX isolate harbors subpopulations with different copy number of the *bla*_NDM-1_. We hypothesize that these bacteria expressed variable levels of the β-lactamase. Indeed, RT-PCR analysis and MIC of KPX cultivated at different imipenem concentrations showed that the repeat sequence impacted on *bla*_NDM-1_ expression and antibiotic resistance phenotype (data not shown).

NDM-1 encodes resistance to all β-lactams except monobactams. However, the plasmid carrying *bla*_NDM-1_ alone does not confer all the antimicrobial phenotypes of KPX. The *E. coli* transformant (ECX-1), which still carries the NDM-1 determinant, remains susceptible to aztreonam and fluoroquinolones. Thus, it is theoretically possible that a superbug such as the *K. pneumoniae* strain reported here may lose some resistance mechanisms on transfer to a new host via the loss of critical sequences from one of the replicons. In the case of the current investigation, no antimicrobial treatment was administered to the patient during hospitalization in Taiwan. As soon as the patient was in a stable condition, he was discharged from the hospital and allowed to stay at home. However, during this time he was continuously monitored for NDM-1 positive bacteria via fecal specimens. These became undetectable after a short period of time. Unnecessary and extended use of the broad-spectrum antimicrobials would probably have prolonged the period of colonization with NDM-1 positive bacteria, and thus increased the chance of possible spread in the hospital and even in Taiwan in general.

By segregating the two plasmids of the original host and by studying the phenotypes of individual plasmid in isolation, we gained insights beyond the DNA sequence. The plasmid pKPX-1 appears to be unstable and non-conjugable; and we found no evidence that it was mobilizable by pKPX-2. Not only were we able to find variation between isolates in the tandem repeats of the NDM-1 region, a large portion of the plasmid sequence was found to be deleted in the plasmid when the *E. coli* transformant was examined (Fig. 2). We confirmed that this truncated form did exist in the original *K. pneumoniae* isolate but it was initially discovered in the pECX-1. We speculate that the truncation of the plasmid and the variable nature of the NDM-1 sequence could be advantageous for KPX to meet the different environmental conditions.

In conclusion, this investigation on the NDM-1-positive *K. pneumoniae* clinical isolate has generated detailed genetic information for its multidrug resistance phenotype. Our study not only provides molecular evidence for tandem duplication of the *bla*_NDM-1_ sequence and a clear demonstration of the heterogeneous nature of carbapenemase gene in a single isolate, but also allows us to present a scheme of genetic recombination through which the bacteria can lose the *bla*_NDM-1_ sequence in the absence of selection pressure. While the clinical implication of this observation is quite clear, the physiological basis underlying the negative impact on fitness by NDM-1 remains unknown and warrants future study taking a systems biology approach.

## Supporting Information

Figure S1 **Identification of the genetic determinants for amikacin resistance.** The predicted amikacin resistance genes are colored orange. These include a gene encoding a putative rRNA methyltransferase, *rmt*, and *aac(6′)-Ib* on pKPX-1, and a *aac(6′)-Ib-cr* gene on pKPX-2. Both ends of the insert in each resistant clone were sequenced, and the sequence pairs mapped back to the complete plasmid sequences. The genomic regions covered by the resistant clones are indicated by dotted lines; and the identification of each clone is given on the left.
(TIF)

Figure S2 **Identification of genetic determinants for chloramphenicol resistance.** The predicted chloramphenicol resistance genes are colored green. Both ends of the insert in each resistant clone were sequenced, and the sequence pairs mapped back to the complete plasmid sequences. The genomic regions covered by the resistant clones are indicated by dotted lines; and the identification of each clone is given on the left.
(TIF)

Figure S3 **The breakpoint at the NDM-1 region.** The remaining sequence of the amplified DNA fragment after losing the NDM-1 region is shown. The arrows indicate the orientation of the repeat-flanking coding sequences (109 and 114). Gray color indicates the 1543-bp repeat identified in the NDM-1 region (as shown in Fig. 3A).
(TIF)
