# Supplementary figures and images for: Copy Number Change of the NDM-1 Sequence in a Multidrug-Resistant Klebsiella pneumoniae Clinical Isolate
Source: PLoS One. 2013 Apr 29;8(4):e62774. doi: 10.1371/journal.pone.0062774 (PMC3639163; doi:10.1371/journal.pone.0062774)

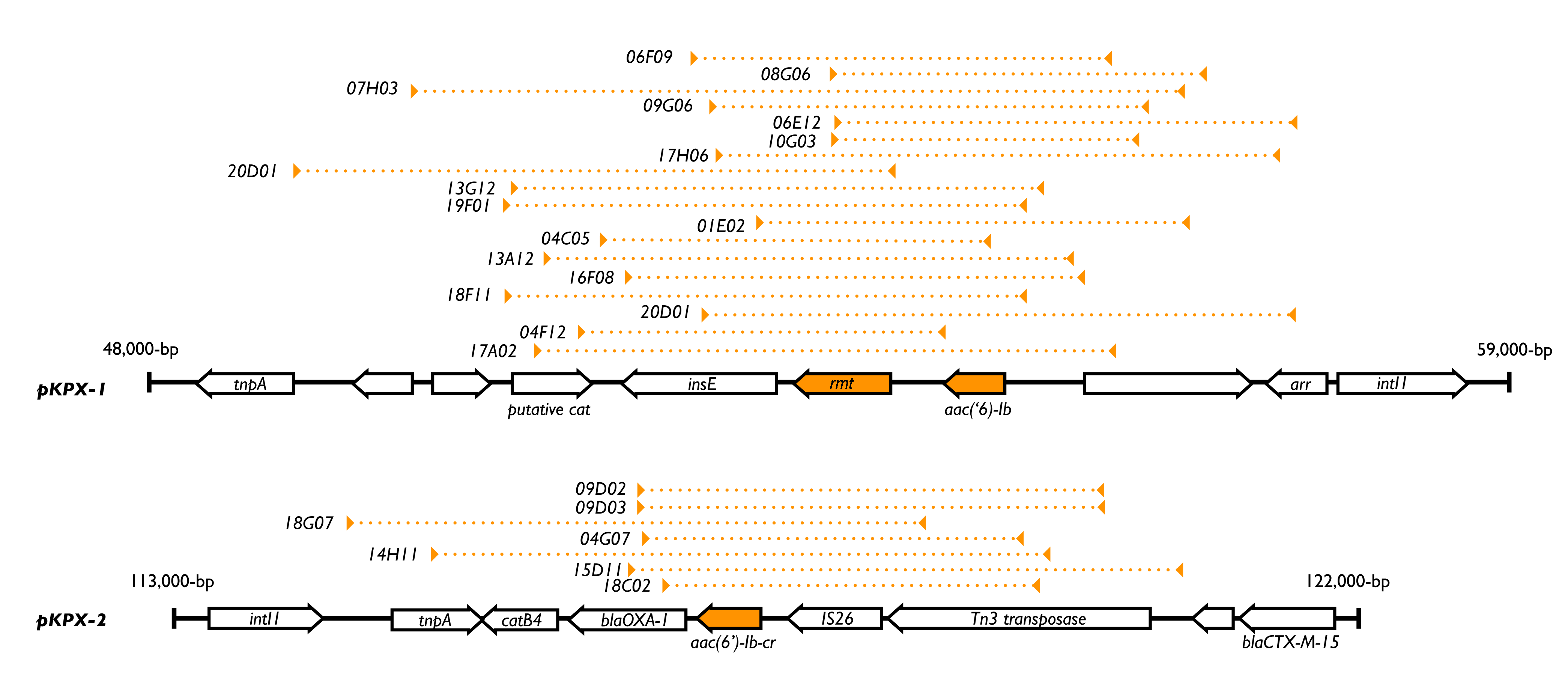

Supplement: Figure S1 — Identification of the genetic determinants for amikacin resistance. The predicted amikacin resistance genes are colored orange. These include a gene encoding a putative rRNA methyltransferase, rmt, and aac(6′)-Ib on pKPX-1, and a aac(6′)-Ib-cr gene on pKPX-2. Both ends of the insert in each resistant clone were sequenced, and the sequence pairs mapped back to the complete plasmid sequences. The genomic regions covered by the resistant clones are indicated by dotted lines; and the identification of each clone is given on the left. (TIF) [file pone.0062774.s001.tif]

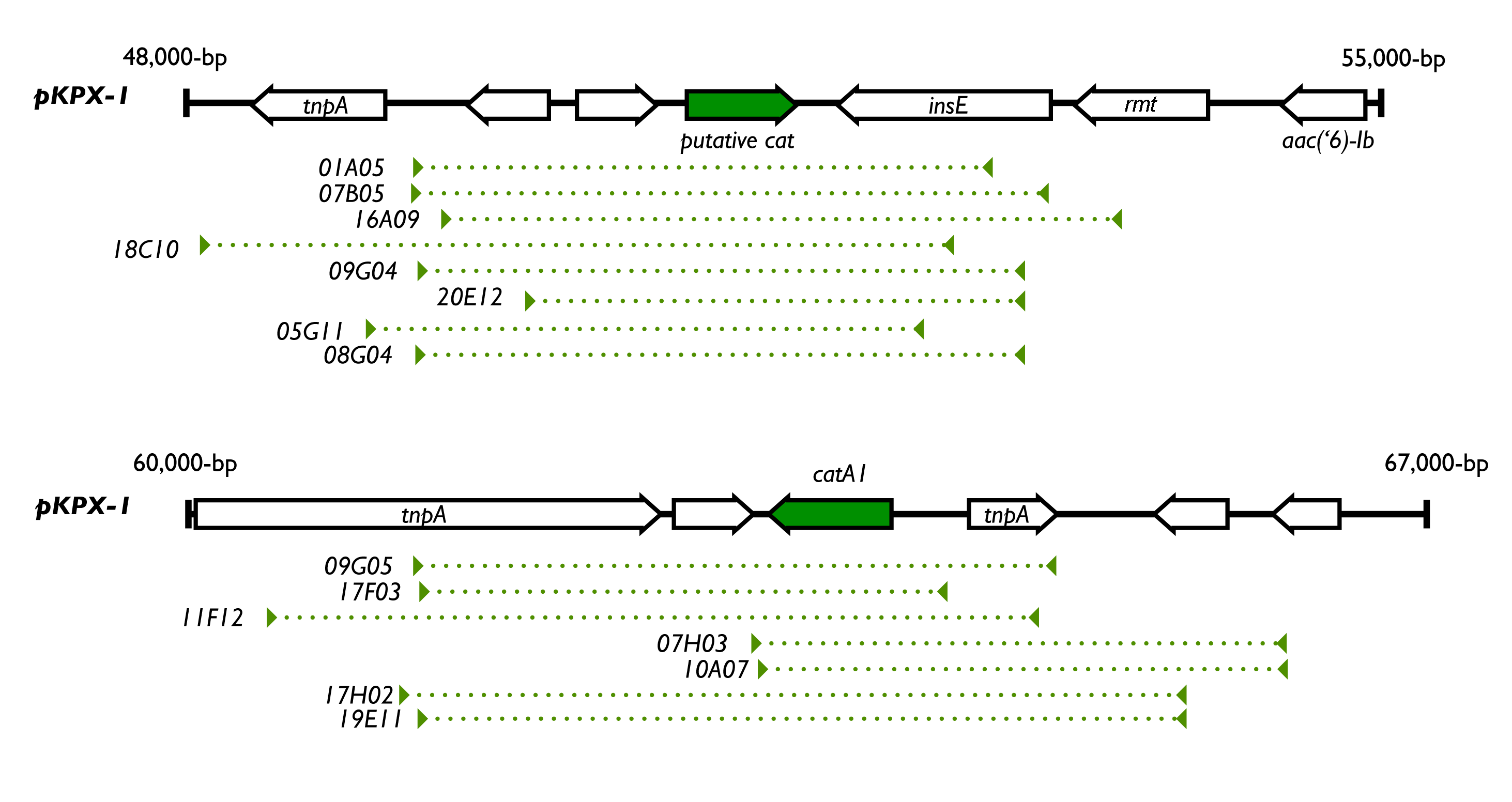

Supplement: Figure S2 — Identification of genetic determinants for chloramphenicol resistance. The predicted chloramphenicol resistance genes are colored green. Both ends of the insert in each resistant clone were sequenced, and the sequence pairs mapped back to the complete plasmid sequences. The genomic regions covered by the resistant clones are indicated by dotted lines; and the identification of each clone is given on the left. (TIF) [file pone.0062774.s002.tif]

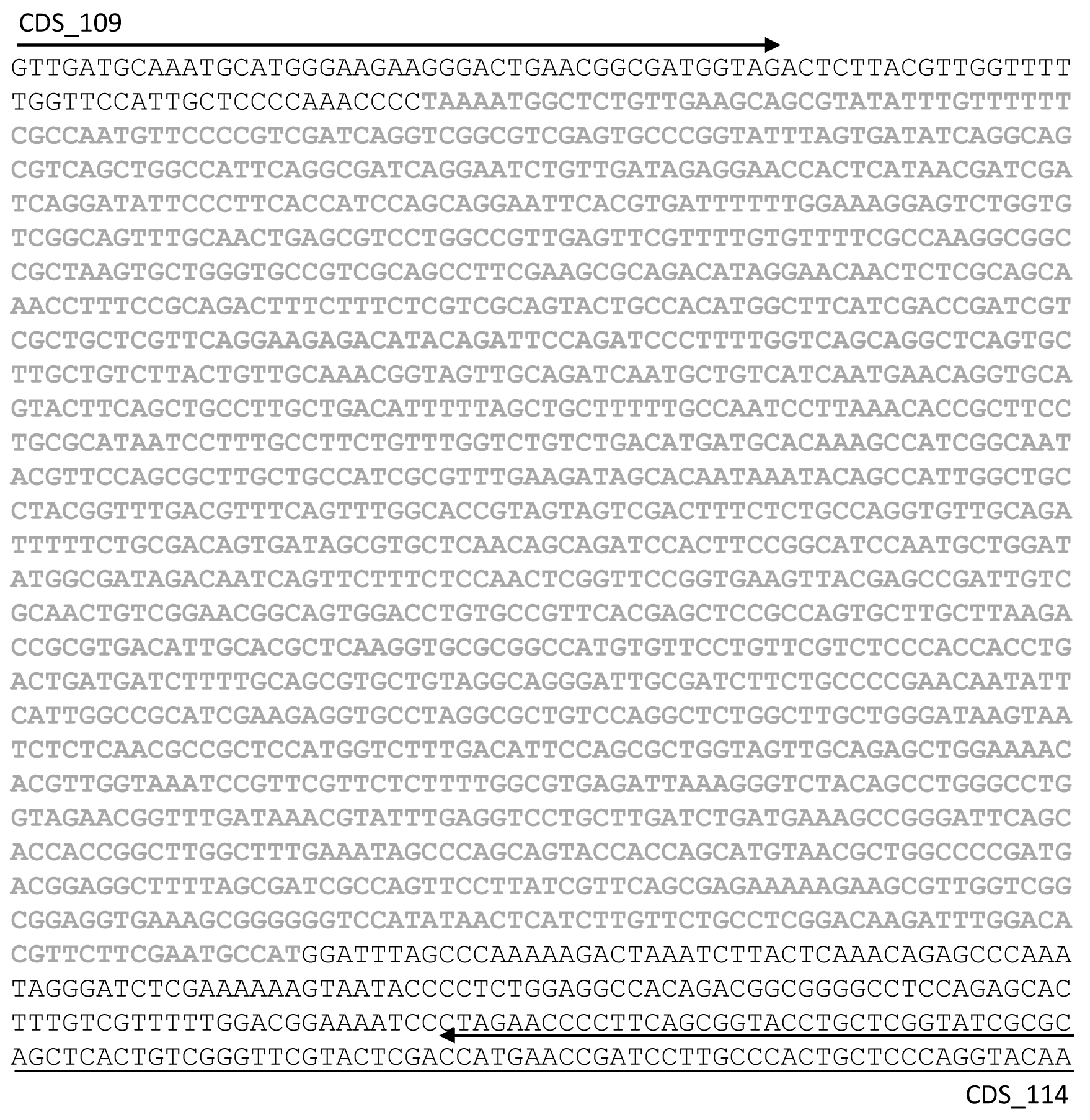

Supplement: Figure S3 — The breakpoint at the NDM-1 region. The remaining sequence of the amplified DNA fragment after losing the NDM-1 region is shown. The arrows indicate the orientation of the repeat-flanking coding sequences (109 and 114). Gray color indicates the 1543-bp repeat identified in the NDM-1 region (as shown in Fig. 3A). (TIF) [file pone.0062774.s003.tif]
